# Finding hotspots: development of an adaptive spatial sampling approach

**DOI:** 10.1038/s41598-020-67666-3

**Published:** 2020-07-02

**Authors:** Ricardo Andrade-Pacheco, Francois Rerolle, Jean Lemoine, Leda Hernandez, Aboulaye Meïté, Lazarus Juziwelo, Aurélien F. Bibaut, Mark J. van der Laan, Benjamin F. Arnold, Hugh J. W. Sturrock

**Affiliations:** 1 Global Health Group, University of California, San Francisco, San Francisco, USA; 2 Ministère de la Santé Publique et de la Population, Port-au-Prince, Haiti; 3 Department of Health, Infectious Disease Office, National Center for Disease Prevention and Control, Manila, Philippines; 4 Programme National de Lutte contre les Maladies Tropicales Négligées à Chimiothérapie Préventive, Ministère de la Santé et de l’Hygiène Publique, Abidjan, Côte d’Ivoire; 5 National Schistosomiasis and STH Control Programme, Ministry of Health, Lilongwe, Malawi; 6 Division of Epidemiology and Biostatistics, University of California, Berkeley, Berkeley, USA; 7 Francis I. Proctor Foundation, University of California, San Francisco, San Francisco, USA

**Keywords:** Medical research, Epidemiology

## Abstract

The identification of disease hotspots is an increasingly important public health problem. While geospatial modeling offers an opportunity to predict the locations of hotspots using suitable environmental and climatological data, little attention has been paid to optimizing the design of surveys used to inform such models. Here we introduce an adaptive sampling scheme optimized to identify hotspot locations where prevalence exceeds a relevant threshold. Our approach incorporates ideas from Bayesian optimization theory to adaptively select sample batches. We present an experimental simulation study based on survey data of schistosomiasis and lymphatic filariasis across four countries. Results across all scenarios explored show that adaptive sampling produces superior results and suggest that similar performance to random sampling can be achieved with a fraction of the sample size.

## Introduction

Recent years have seen considerable success towards control and elimination of a range of globally important infectious diseases. For many of these diseases, decisions relating to interventions are made across administrative units. For example, decisions about where to conduct mass drug administration (MDA) campaigns for neglected tropical diseases (NTDs) are made at an implementation unit (IU), typically the district or sub-district level^1^. A similar approach is typically taken in the control and elimination of malaria, where entire districts or sub-districts may receive insecticide treated nets or indoor residual spraying where others do not.

For NTDs, decisions relating to MDA are based on infection prevalence estimates at the IU level obtained from cross sectional surveys. Where IU level prevalence exceeds a threshold, the entire IU is treated^1^. Where prevalence does not exceed this threshold, the IU does not qualify for MDA and no individuals in that area are treated. For example, for schistosomiasis, current guidelines recommend that MDA is conducted in areas where prevalence is greater than 10%, whereas for soil-transmitted helminths, this threshold is 20%^1^.

While operationally straightforward, this approach ignores any within IU heterogeneity. In many instances, districts with prevalence below the threshold that triggers intervention contain a number of villages with active transmission^2^. Modeling and intuition therefore suggest that as disease transmission declines, moving away from decision making at coarse scales towards a more targeted approach is more cost-effective^3^. Such targeting is predicated on sufficiently accurate information on the location of sites with an infection prevalence above a policy relevant threshold, from hereon referred to as *hotspots*.

Missing hotspots could cause setbacks for elimination efforts. Hence, various approaches to identify them have been proposed. Variations of contact tracing, whereby testing is targeted at families and neighbours of individuals found positive during surveys or routine surveillance, have been explored for a number of diseases including schistosomiasis^4^, lymphatic filariasis^5^ and malaria^6,7^. Such approaches can, however, be expensive and can still fail to identify hotspots if positive individuals from those communities are not identified by the initial surveys.

An alternative approach is to use less costly survey methods to sample a higher proportion of locations than would otherwise be possible. Techniques such as lot quality assurance sampling, a method designed to minimize sampling effort in order to categorize outcomes over a given population, is one such approach and has been used to identify hotspot communities for schistosomiasis^8,9^. Similarly, school-based questionnaires relating to blood in urine and eye worm occurrence, have been used to map urinary schistosomiasis^10–12^ and loiasis^13,14^ respectively. These methods are inherently noisy as they only allow measurement of proxies of infection and can suffer from issues of recall.

Another approach to mapping hotspots, which reduces the need to sample a large fraction of the population, is using geospatial modeling. Climatological, environmental and ecological layers can help predict the spatial distribution of many infectious diseases. Furthermore, above and beyond patterns that can be explained by these layers alone, disease outcomes often display some spatial structure, with neighbouring values being correlated due to shared characteristics and transmission. This spatial structure means that information from one site provides information about neighbouring sites. Over the past decade, the ability to predict pathogen infection prevalence across entire regions based on survey data and relationships using geospatial modeling has improved considerably^15–17^. These advances in geospatial modeling have opened the door to more targeted approaches, potentially allowing decisions about treatment to be made with higher precision and granularity.

Despite these advances, surprisingly little attention has been paid to optimizing the survey design for risk mapping efforts. Evidence from other fields has shown that random sampling is suboptimal for spatial prediction^18–21^. For lymphatic filariasis, a grid sampling approach has been proposed as a mechanism to allow for more efficient spatial interpolation^22,23^. Diggle and Lophaven^24^ propose the use of grid sampling supplemented with clusters of close pairs of points which is useful when estimates of Kriging (covariance) parameters are required^24^. Simulation studies also suggest that this design provides a cost-effective approach to mapping schistosomiasis^3^. Similarly, Fronterre et al.^25^ show that spatially regulated surveys, in combination with spatial modeling, can reduce the sample size required to estimate IU level prevalence.

Recent studies by Chipeta et al.^26^ and Kabaghe et al.^27^ propose the use of spatially adaptive designs that leverage information from prior data to inform the locations of future sampling sites to minimize prediction error. Using malaria as an example, results from simulations and field studies show that adaptive spatial designs can be used to produce more precise predictions of infection prevalence using geostatistical modeling^26^.

Building on the adaptive spatial sampling approach, we incorporate ideas from Bayesian optimization theory^28,29^ to propose an adaptive spatial sampling approach optimized to identify hotspot communities.

## Results

We compared the performance of two approaches for selecting survey sites: random sampling (RS), where sites are chosen randomly; and adaptive sampling (AS), that follows the acquisition function of Eq. (6). The underlying statistical model is the same in both cases (see Eqs. (1)–(2)). The initial dataset ${\mathscr {D}}_0$ is also the same in both cases (see Table 2 lines 7 and 8). Hence, the variations in the performance with respect to the predictions based on ${\mathscr {D}}_0$ depend only on the mechanism of selecting the new survey locations ${\mathscr {A}}_1, {\mathscr {A}}_2, \ldots$. Adding measurements at new locations improved out-of-sample sites classification under both sampling approaches. However, across the four scenarios tested we observed that adaptive sampling was consistently superior to random sampling in terms of accuracy, positive predictive value and sensitivity.

**Figure 1:**
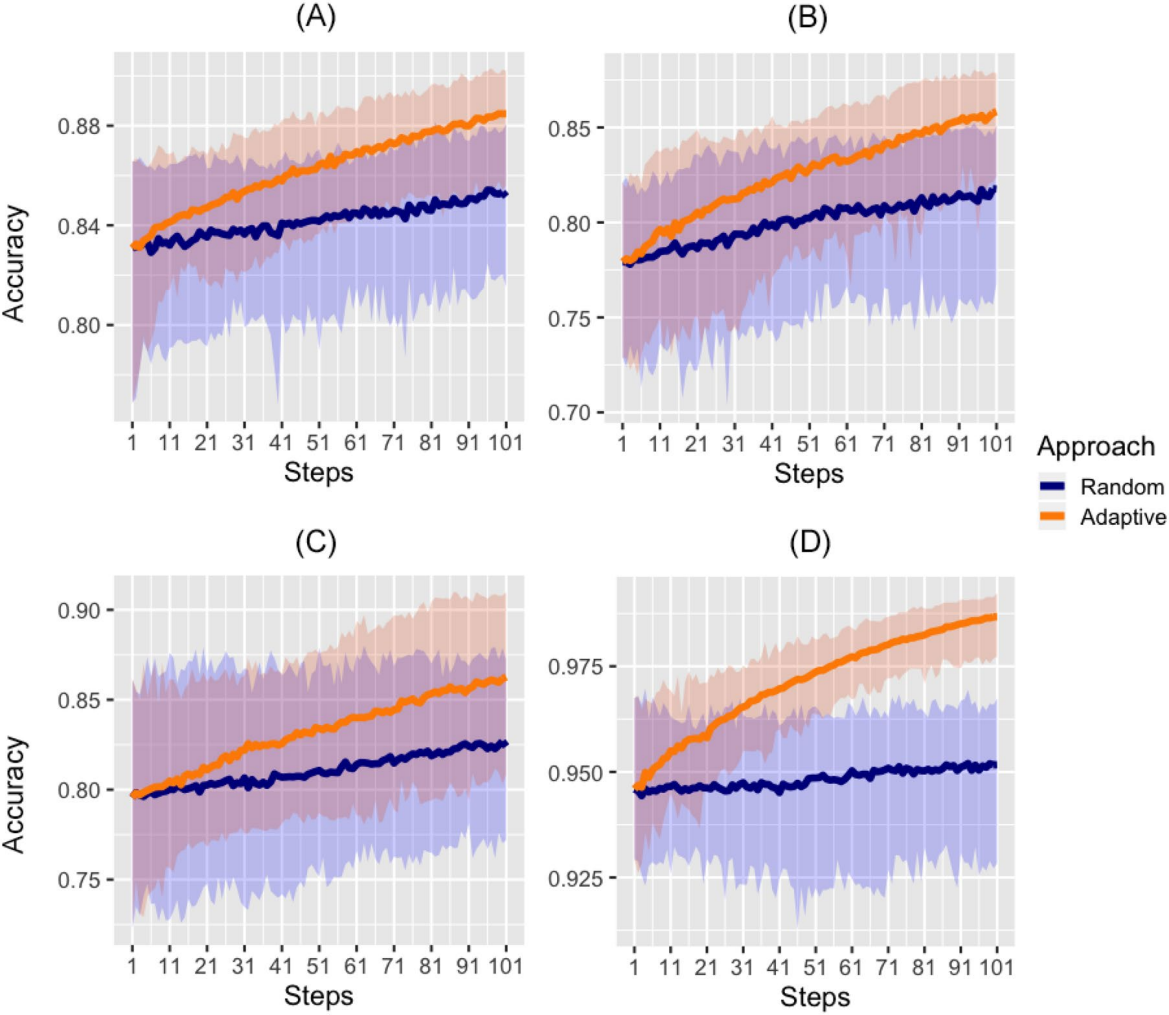
Out of sample accuracy (batch size = 1). The solid line represents the average value across 50 repetitions. The shaded area represents the 2.5% and 97.5% quantiles of the values observed across all 50 repetitions at each step. Note that step 1 here refers to the initial random sample of 100 sites. (**A**) Côte d’Ivoire ($\vartheta =10\%$). (**B**) Malawi ($\vartheta =10\%$). (**C**) Haiti ($\vartheta =2\%$). (**D**) Philippines ($\vartheta =2\%$).

This confirms that under adaptive sampling each new batch of locations leads to a better classification of the unmeasured sites. Figure 1 shows the accuracy computed at each step in the four country scenarios using a batch of size 1. Note that when selecting a batch of size 1, the adaptive design does not take into account the exploration component. In this case the new location suggested is the one that maximizes entropy.

**Figure 2:**
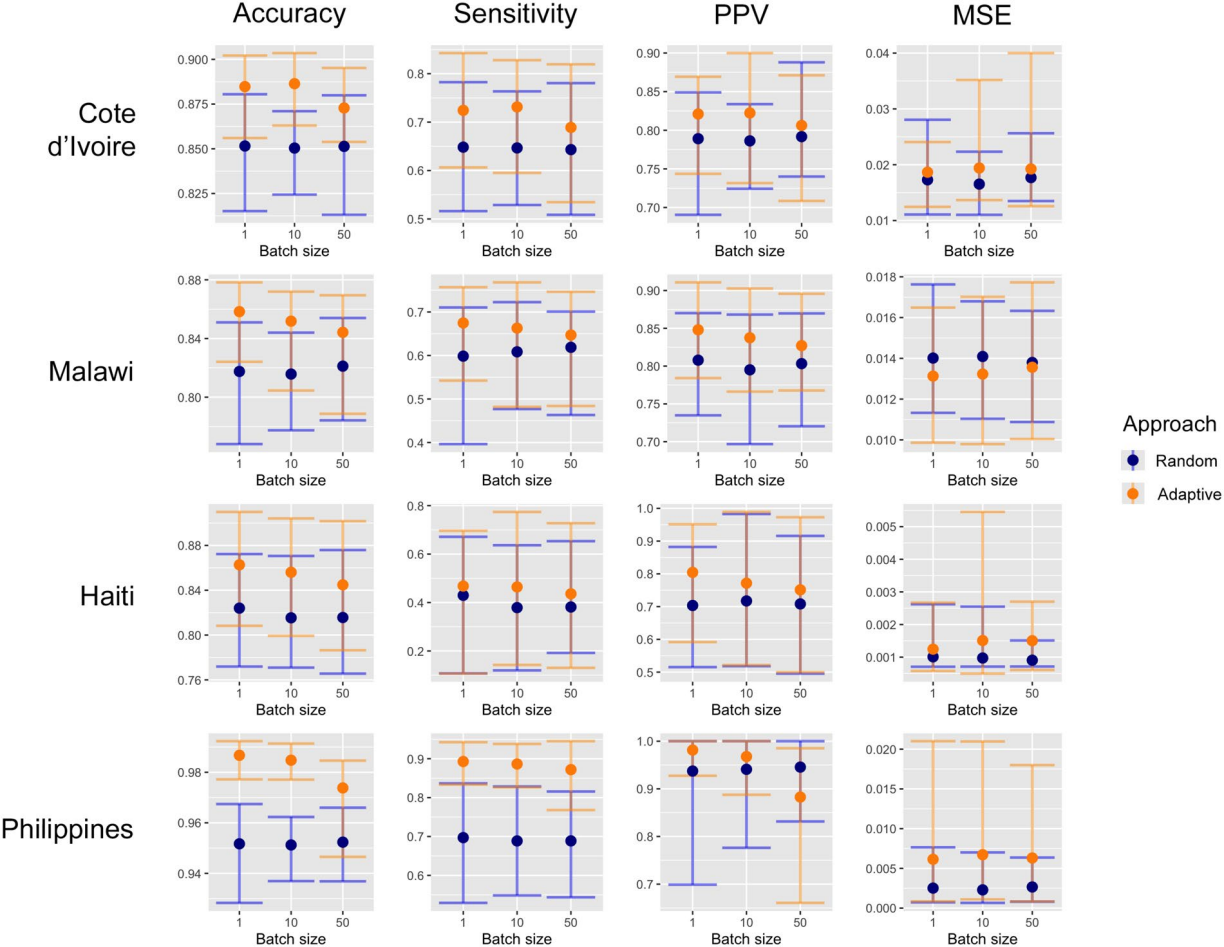
Summary of validation statistics. Metrics computed after adding 100 new samples in batches of 1, 10 and 50 sites. Dots represent the mean and whiskers represent the the 2.5% and 97.5% quantiles of values observed across all 50 repetitions. The thresholds used to define a hotspot are: $\vartheta =10\%$ in Côte d’Ivoire and Malawi and $\vartheta =2\%$ in Haiti and Philippines.

Figure 2 shows a summary of the validation statistics after adding 100 new samples, using different batch sizes (1, 10 and 50), across the four scenarios. The results show that adaptive sampling produces superior accuracy, sensitivity and PPV across every scenario, metric and batch size except in the Philippines where an adaptive approach with a batch size of 50 produced inferior PPV. Better performance across all metrics translates into a smaller number of false positives and a improved identification of hotspots in locations that have not been visited yet. In contrast to the validation statistics discussed above, MSE (bottom row) is lower across all scenarios when random sampling was employed, except in Malawi where adaptive sampling produced lower MSE.

**Table 1:** For random design RS with sample size of 100, we show the sample size needed to achieve a similar accuracy using an adaptive design AS.

Country	$\Vert {\mathscr {A}}_i\Vert$	Num. obsv.		Accuracy (%)		PPV (%)		Sensitivity (%)		MSE ($\times 10^{-4}$)	
		RS	AS	RS	AS	RS	AS	RS	AS	RS	AS
Côte d’Ivoire	1	100	27	85.2	85.3	64.9	78.6	64.8	65.1	17.3	20.5
	10	100	30	85.0	85.3	78.6	78.7	64.7	65.2	16.5	20.4
	50	100	50	85.1	85.5	79.2	78.6	64.3	65.5	17.7	20.0
Malawi	1	100	36	81.8	81.9	80.8	80.6	59.9	59.4	14.0	14.9
	10	100	40	81.6	82.0	79.5	80.5	60.8	59.7	14.1	15.0
	50	100	50	82.1	82.1	80.3	80.3	61.9	60.4	13.8	14.5
Haiti	1	100	31	82.4	82.6	70.3	75.7	43.0	35.0	1.0	1.4
	10	100	30	81.5	81.6	71.7	71.0	38.9	36.4	1.0	1.5
	50	100	50	81.5	82.3	70.8	70.5	38.1	39.8	0.9	1.5
Philippines	1	100	7	95.2	95.2	93.7	94.4	69.7	67.7	2.5	4.3
	10	100	10	95.1	95.2	94.1	93.6	68.9	68.6	2.3	5.1
	50	100	50	95.2	95.6	94.6	85.0	68.9	79.8	2.7	5.5

Additional validation statistics: PPV, sensitivity and MSE are also shown. Along the rows, results are shown per country and batch size $\Vert {\mathscr {A}}_i\Vert$.

At larger batch sizes there were smaller differences between random and adaptive sampling in terms of accuracy, PPV and sensitivity (Fig. 2). There are two ways of interpreting this result. One interpretation is that when the batch is large enough, random sampling provides a good coverage of the sampling universe negating the need for a trade-off between exploitation and exploration. The more locations in the batch the more redundant the information they provide, regardless of how they are chosen. A second interpretation is that the adaptive sampling design is more efficient and therefore requires smaller sample sizes to achieve the same results of a larger random sample. Table 1 illustrates this and shows the number of sample points needed when using adaptive sampling to achieve the same accuracy of random sampling with a sample size of 100 locations. For batches of size 50, adaptive sampling produced at least the same level of accuracy with just half the number of additional points across all scenarios. This difference becomes larger for smaller batch sizes (1 or 10). For batch sizes of 10, adaptive sampling required 10–40% of the sample size to achieve the level of accuracy achieved with 100 additional randomly selected sites and for batch sizes of 1, only 7–36% was required. With such sample sizes the adaptive sampling also achieved similar levels of sensitivity and PPV but higher MSE.

## Discussion

The identification of disease hotspots is an increasingly important public health problem. This is particularly true in disease elimination settings, where transmission is rare and typically focal. Numerous examples illustrate the use of geospatial modeling to predict hotspots, but very little attention has been given to the optimal survey design for such modeling efforts. Here, using simulation studies based on schistosomiasis and lymphatic filariasis survey data, we described a novel, spatially adaptive approach and demonstrate the superiority of this approach at identifying hotspots compared with the standard approach to surveys based on purely random sampling.

Results showed that across all batch sizes investigated, adaptive approaches produced higher levels of accuracy, sensitivity, and PPV compared with random sampling. Yet, the superiority of an adaptive approach declined with larger batch sizes. With a batch size of 1, the adaptive approach has an opportunity to identify the optimal next location to survey in the presence of all available data. In contrast, with larger batch sizes, the impact on predictions of each adaptively sampled location is not known until all locations in the batch are sampled and the model updates.

The use of an adaptive approach only produced marginal gains in accuracy (2–4%) after adding 100 sites to the initial sample, but this could represent hundreds of locations when applied at a country scale. Perhaps more importantly, however, adaptive sampling was more efficient in terms of achieving a given level of accuracy with a far smaller sample size. As outlined in Table 1, across all scenarios explored, adaptive sampling was able to achieve the same level of accuracy and sensitivity to that achieved by adding 100 locations randomly with between 7 and 50% the sample size. These results demonstrate that an adaptive spatial sampling approach has the potential to substantially reduce the resources required to ensure hotspot locations receive treatment, while maintaining similar rates of false positives. In control and elimination settings, an operationalized adaptive spatial sampling approach for several years could render non-negligible improvements in cost-effectiveness. Further simulation studies could be used to help determine the magnitude of such benefits in cost-effectiveness.

It should also be pointed out that in almost all settings the mean squared error estimates were higher for adaptive approach (Fig. 2, Table 1). This illustrates the fact that optimizing a design for one goal, here hotspot classification accuracy, leads to compromising other goals (e.g. precision in the prevalence estimates). Where the goal is to produce the most precise prevalence estimates at any given location, using adaptive approaches based on prediction variance as opposed to entropy would be more appropriate^26^.

While this approach was demonstrated for two diseases only, it could be used to support the identification of hotspots of any binomial outcome. This includes prevalence of infection of other infectious and non-infectious diseases, particularly those that display strong spatial correlation. Vector-borne diseases, such as malaria, onchocerciasis and loiasis would certainly fall into this category given the association between disease transmission and ecological and environmental conditions. While it is likely that such spatial correlation will be masked following several years of intervention, evidence suggests that residual hotspots still occur^2,30^. In addition to identifying hotspots of infection, this approach also has potential utility for identifying *cold spots* in intervention coverage, such as pockets of undervaccination^31^. While this would likely require use of different covariates related to intervention access, such as distance to roads, population density and poverty, the statistical problem is analogous.

In principle, in the context of schistosomiasis and lymphatic filariasis, including additional relevant covariates could improve the spatial model predictions. Among others, these could include information on intervention coverage, population density, poverty, housing type and soil type. For the purpose of this simulation study, we opted to use WorldClim data as the focus was on the marginal improvement of using an adaptive sampling approach over a random sampling, as opposed to identifying the optimal model and covariates with which to predict infection.

While we used a combination of random forest and model-based geostatistics to produce posterior prevalence estimates, the general adaptive sampling scheme we have proposed would work for any suitable modeling approach that produces posterior estimates with which to estimate exceedance probabilities. Combining random forests with other base learners such as generalized additive models and support vector machines may lead to improvements over using random forest alone. Such a ‘super learner’ approach to ensemble modeling, based on minimizing cross-validation error, may help to address any issues of model misspecification which if not properly addressed could lead the adaptive design to become overly confident about choice of sampling location. Super learning has been used across a range of other statistical problems including causal inference and prediction^32–34^. Furthermore, the super learner approach can be extended to be ‘online’ whereby models are updated rather than refit from scratch, yielding computational benefits^35^.

Similarly, an underlying binomial model is not essential to the methodology described here. What is important is the spatial correlation component in which the exploration rule is based. For example, this methodology could work in a Poisson setting, for some definition of hotspot based on a threshold incidence or numbers of cases.

A further potential extension of this work would be to incorporate covariates into the adaptive sampling algorithm. The approach outlined here attempts to reduce redundancy when selecting sampling locations by prioritizing uncertainty across geographic space. However, the uncertainty of the model does not only depend on the geographic space encoded through the Matérn kernel. Uncertainty is also dependent on the remaining features (covariate values). In this application, a good spread of points in geographic space is likely to achieve a good spread of points in the remaining features, as their values are determined by location. In applications where the covariates are less influenced by geography it may be necessary to optimize the design for the uncertainty in the whole feature space. Not doing so may lead to a design that is no better than random. The most straightforward way to implement this extension would be to model the covariates with another kernel (Matérn, linear or any other considered adequate). In such a model the term $\mathbf{x} _i^\top {\varvec{\beta }}$, in Eq. (2), would be substituted by another Gaussian process $f( \mathbf{x} _i)$ that depends on the new kernel. Equation (4) would remain the same, but this time *K* would in fact be the sum of two kernels: the Matérn that models geographic space and the new kernel that models the remaining features.

Another possible extension of this methodology is applying it to cases where the classification of interest is not binary. For example, for schistosomiasis, MDA is recommended once per year in areas where prevalence is $>10\%$ and $<50\%$ and twice per year in areas where prevalence is $>50\%$^1^. As estimation of entropy is not restricted to binary classification problems, adapting the approach to such a setting is straightforward assuming it is possible to produce probabilistic classifications from the underlying model.

This study had a number of limitations. Firstly, the adaptive sampling approach described requires a georeferenced set of candidate sampling locations. Complete georeferenced lists of settlements are, however, often not available. In the absence of such data, there are several options available. Georeferenced locations could be extracted and compiled from open sources, such as OpenStreetMap, Geonames and openAFRICA. Alternatively, village locations can be derived from gridded population data using the approach described here (see Supplementary Information) or using alternative approaches as suggested by Thomson et al.^36^.

A second limitation is that we did not consider the temporal aspect of adaptive surveys. In reality, there may be a time lag between the date at which survey data are available and when adaptive surveys take place. Similarly, prior survey data may have been collected over multiple time periods. To address this issue it would be possible to extend the spatial model used, to a spatio-temporal model. Hotspot probabilities could then be forecast from the historic data to the time point at which adaptive surveys are to take place. Additionally, there may be value in using temporally dynamic covariates as opposed to static, long-term averages as used here.

A third limitation was that we defined a site as a hotspot if there was at least a 50% chance that prevalence exceeded the relevant threshold. In some cases, programs may a priori wish to define hotspots more conservatively by classifying sites as hotspots with smaller probabilities (e.g. $>10\%$ chance a site is a hotspot). While the methodology would not change, such an approach would have a large impact on the performance of the classifications, increasing sensitivity, but decreasing positive predictive value. In such cases, it may also be useful to modify the acquisition function.

A fourth limitation of this study is that we used a single acquisition function. In the acquisition function we used, the exploration component has an increasing concave weight as more locations are added to the new batch. This assumption, or the specific shape of this weight, could be substituted for an alternative. Also, the utility function, defined here as entropy, could be modified depending on the goal pursued. For example, a program interested in targeting sampling efforts at hotspots, instead of achieving a better binary classification, could use the probability of a location being a hotspot as the utility function. Such an approach would be suitable for situations where testing is required before an intervention/treatment is administered. This approach may also be useful for surveys whose goal is to determine freedom from infection^37,38^.

A fifth limitation stems from the simulated nature of the experiments. The strength of a simulated approach is that multiple experiments can be conducted without the need for expensive field validation studies. On the basis of these results, a valuable next step would be to conduct field studies comparing random to adaptive designs. Such studies would also allow an exploration of some of the more logistical elements and constraints and using an adaptive approach.

This study has demonstrated the value in adopting an adaptive approach to surveys designed to identify disease hotspots. Results show that a spatially adaptive sampling approach produced consistently superior accuracy in hotspot classification over a random sampling approach, and could dramatically lower the resources requirements to conduct surveys whose goal is to detect disease hotspots.

## Methods

### Spatial model

To predict the probability that a given site (e.g. a village or other type of settlement) is a hotspot or not, and to guide adaptive sampling schemes, requires fitting a spatial model to observed data. As a reminder, here a hotspot is defined as a location where infection prevalence is greater than a defined threshold. We assume that an initial representative population sample exists to allow a model to be fit. If this is not the case, a randomly sampled set of measurements would be one option, although there may be superior approaches, particularly if data relating to the expected spatial structure or covariate values at candidate survey sites exist^24,39,40^.

There are a range of different modeling approaches available to predict prevalence at unsurveyed sites. Here, we use a combination of machine learning and model-based geostatistics^15,41^.

Let ${\mathscr {B}}$ be a region (e.g. a country) where we are interested in determining if a set of sites are hotspots or not. As mentioned above, it is assumed that an initial dataset from which we can estimate the overall prevalence exists. Say we have the dataset ${\mathscr {D}}_0 = \{ { \mathbf{s} _i, n_i, y_i, \mathbf{x} _i} \}_{i=1}^{m_0}$, where $\mathbf{s} _i$ are the GPS coordinates that describe the location of a site of interest, $n_i$ is the number of people tested in such site, $y_i$ are the number of positive cases out of $n_i$ and $\mathbf{x} _i$ are other features associated to the site, like elevation, distance to water bodies or average temperature; $m_0$ is the total number of observations. Given these data we can model the prevalence in ${\mathscr {B}}$ as a spatially continuous process given by

1 $$\begin{aligned} y_i\sim & {} \text{ Binomial }(n_i, \theta _i), \end{aligned}$$

2 $$\begin{aligned} \text {logit}(\theta _i)= & {} \mathbf{x} _i^\top {\varvec{\beta }} + f( \mathbf{s} _i) + \mathbf{e} _i; \end{aligned}$$

where ${\varvec{\beta }}$ are a set of real parameters and *f* is a spatially correlated random effect using a Matérn correlation function (see Supplementary Information, Eq. (7)) and $e_i$ is a residual independent error term.

Instead of including linear covariate effects, we first fit a random forest model using 20-fold cross validation using all the covariates, excluding latitude and longitude. For each observation, we then have a cross-validated prevalence prediction (from hereon termed *out-of-sample predictions*). Additionally, we fit a random forest using all observations and use this model to predict to all observation and prediction points (from hereon termed *in-sample predictions*). Out-of-sample predictions from the random forest are then included as a single covariate in the geostatistical model described by Eq. (2).

When making predictions, in-sample predicted prevalence values from the random forest using all observations were used as the covariate at each prediction point. While this model allows us to predict prevalence across the continuous region ${\mathscr {B}}$, in this case we are only interested in predictions at the location of human settlements. Here, we denote these discrete locations as ${\mathscr {S}} \subset {\mathscr {B}}$.

In addition to obtaining estimates of predicted prevalence, the model described above allows us to simulate a posterior distribution of prevalence values at each cluster which can be used to estimate the *exceedance probabilities*, i.e. the probability that prevalence $\theta _i$ at location $\mathbf{s} _i$ is above a given threshold $\vartheta$.

### Adaptive sampling

#### Exploitation

The goal we seek when using adaptive sampling or adaptive design is to leverage the information available and select the *optimal* sampling locations to improve our statistical inference^28,29^. The criteria to define what is optimal depends on what quantity is to be estimated. Hence, it is first necessary to define an objective or utility function, i.e. the measure by which we evaluate the performance of any given design. For situations where the goal is to produce as precise predictions as possible over the study region, measures such as average prediction variance is a sensible option^26^. If, however, the goal is to find hotspots, we are less interested in the precision of our estimates and should be focused on minimizing hotspot classification error from our model. Put another way, we wish to increase our confidence that the prevalence at any given location is above or below the predefined threshold. A measure that fits naturally into this framework is *Shannon entropy*. Shannon entropy measures the uncertainty of a random variable based on its probability distribution^42^. Let $\vartheta$ be the relevant threshold. Given the model described in Eq. (1), for every $\mathbf{s} _i \in {\mathscr {S}}$ we can estimate its probability of being a hotspot $p(\theta _i > \vartheta | {\mathscr {D}}_0)$. Then the entropy value at such location regarding it being a hotspot or not is defined as

3 $$\begin{aligned} \begin{aligned} H(\theta _i | \mathbf{s} _i, {\mathscr {D}}_0)&= - p(\theta _i> \vartheta | {\mathscr {D}}_0) \log _2 p(\theta _i > \vartheta | {\mathscr {D}}_0) \\&\quad - p(\theta _i \le \vartheta | {\mathscr {D}}_0) \log _2 p(\theta _i \le \vartheta | {\mathscr {D}}_0). \end{aligned} \end{aligned}$$

Locations with exceedance probabilities of 0.5 (i.e. $p(\theta _i > \vartheta | {\mathscr {D}}_0) = \frac{1}{2}$) are the most uncertain and have an entropy value of one. On the contrary, the more certainty in the event (i.e. exceedance probabilities close to 0 or 1), the entropy gets closer to 0. By targeting high entropy values, sampling is focused on those sites with highest classification (hotspot or not) uncertainty.

#### Exploration

**Figure 3:**
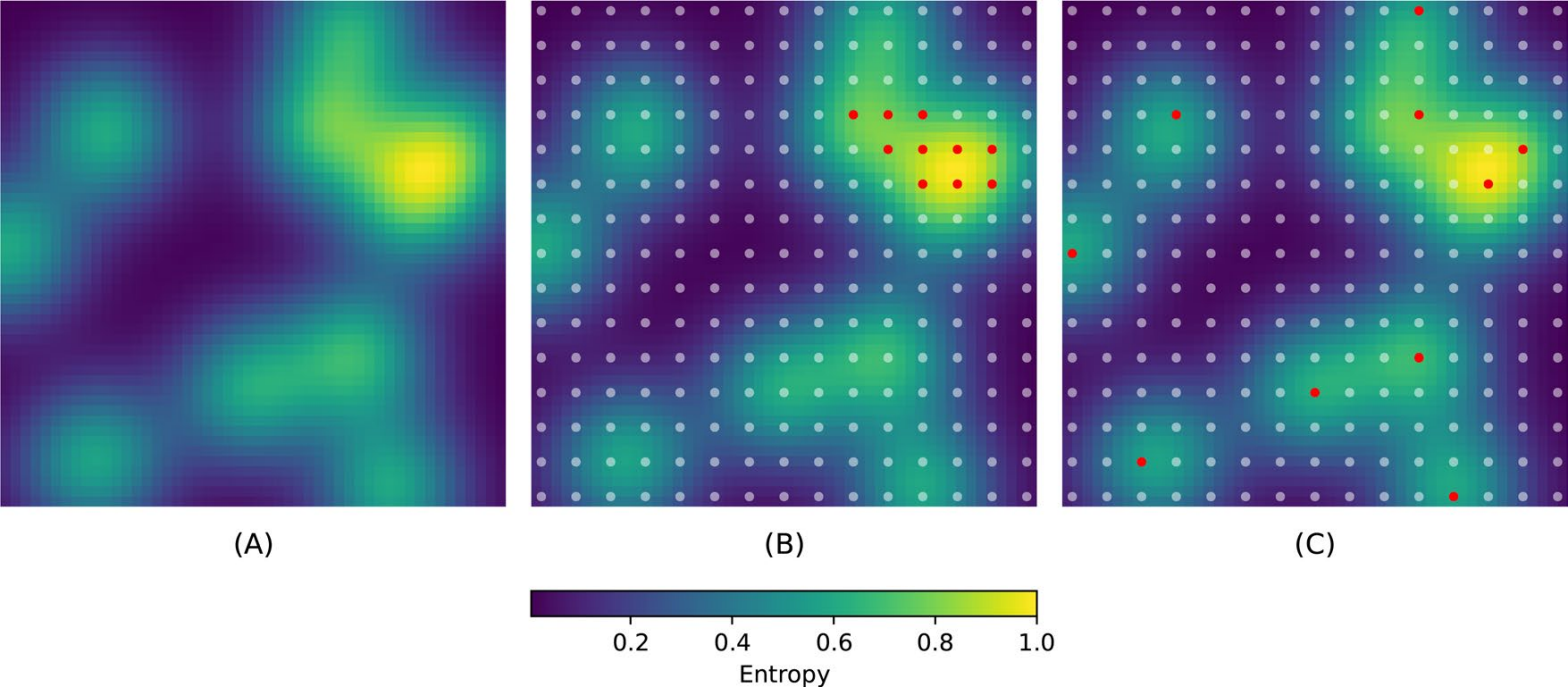
Exploration-exploitation trade-off. (**A**) Spatially correlated uncertainty. (**B**) Batch selected (red dots) by using the greedy approach of targeting the highest values of uncertainty. (**C**) Batch of locations selected (red dots) using the acquisition function described in Eq. (6).

Giving preference to locations with higher uncertainty is intuitively more efficient than a uniform random selection, but choosing the design based only on entropy values (Eq. (3)) may not be efficient because prevalence is usually a spatially correlated process. For example, see Fig. 3 panel A, where we show a simulated field of uncertainty where values are spatially correlated. Since locations with high uncertainty can be expected to be clustered together, by defining a batch of sample points based only on $H(\theta _i | \mathbf{s} _i, {\mathscr {D}}_0)$ we may end up selecting locations that are very close to each other. However, such an approach leads to redundancy, as taking a measurement at one location also provides information about neighboring locations due to the spatial correlation present. In Fig. 3 panel B, we choose the 10 locations (red dots) with highest uncertainty values from a grid of $15 \times 15$ potential locations (white dots). The Figure demonstrates how this greedy approach can result in poor coverage of the field.

It would be preferable to sample high entropy points, while ensuring a good spread of points across the study area to avoid redundancy. This allows a balance between exploitation (i.e. targeting high values of $H(\theta _i | \mathbf{s} _i, {\mathscr {D}}_0)$) and exploration (i.e. spread batch locations in ${\mathscr {B}}$)^43^. If in Eq. (2) we assume that *f* is a multivariate Gaussian with spatial covariance $\mathbf{K} ( \mathbf{s} _i, \mathbf{s} _j)$, then the average amount of information contained in a batch of locations ${\mathscr {A}} = \{ \mathbf{s} _1, \ldots , \mathbf{s} _{m_1}\}$ is given by the joint *differential entropy*

4 $$\begin{aligned} h( \mathbf{f} _{{\mathscr {A}}}) = \frac{1}{2} \log (2\pi e)^{m_1} | \mathbf{K} _{{{\mathscr {A}}}{{\mathscr {A}}}} |, \end{aligned}$$

where $$\mathbf{f} _{{\mathscr {A}}} = (f( \mathbf{s} _1),\ldots , f( \mathbf{s} _{m_1}))^\top$$ and $$\mathbf{K} _{{{\mathscr {A}}}{{\mathscr {A}}}} = [ \mathbf{K} ( \mathbf{s} _i, \mathbf{s} _j)]$$.

The differential entropy is the continuous case of the Shannon entropy introduced before^42^. A low value of $h( \mathbf{f} _{{\mathscr {A}}})$ implies that the random variable $\mathbf{f} _{{\mathscr {A}}}$ is confined to a small volume, whereas a large value of the differential entropy implies a that the variable is widely dispersed. Given a batch size, by choosing the elements in it that maximize the differential entropy, we would be maximizing the average information content of the batch with respect to the random field *f*. Finding the batch with highest information content is a problem of combinatorial complexity. However an exact solution is not needed^44^. A approximate solution can be found through a sequential approach that at step *t* selects the new element of the batch according to

5 $$\begin{aligned} \mathbf{s} ^*= {\text {argmax}}_{ \mathbf{s} \in {\mathscr {S}}} h\left( \mathbf{f} _{{\mathscr {A}}_{t-1} \cup \{ \mathbf{s} \}} \right) . \end{aligned}$$

#### Trade-off

Once we have a utility function and a rule for exploration, we only need to define a trade-off strategy between exploration and exploitation that helps us select a batch of new survey locations. In Bayesian optimization, this strategy is defined by the *acquisition function*^45,46^. Notice, however, that our setting is simpler than the usual setting for Bayesian optimization, where evaluating the utility function is considered to be expensive and the exploration sites could be infinite. In this application we assume a finite set of potential survey locations, as they represent villages or some type of human settlements. Also, in all of these locations we have a measurement of our utility function through the posterior distribution of $\theta$.

As trade-off strategy we define the step-wise algorithm that combines Eqs. (3) and (5), so that at step *t* the new element in the batch is chosen according to

6 $$\begin{aligned} y( \mathbf{s} _t) = {\text {argmax}}_{ \mathbf{s} \in {\mathscr {S}}} \{ H(\theta | \mathbf{s} , {\mathscr {D}}_0) + \sqrt{\log t} \times h\left( \mathbf{f} _{{\mathscr {A}}_{t-1} \cup \{ \mathbf{s} \}} \right) \}. \end{aligned}$$

In the expression above we are explicitly defining *y* as a function of $\mathbf{s}$ to emphasize that we are interested in selecting survey locations. By using this acquisition function we induce batch locations to be spatially scattered and therefore achieve a better exploration. In Fig. 3 panel C, we show a batch of 10 locations (red dots) chosen according to Eq. (6). The locations selected are not the ones with the overall highest uncertainty, but the ones with the highest uncertainty within a neighborhood. This approach allows targeting high entropy values, while reducing information redundancy and exploring the region of interest.

The acquisition function in Eq. (6) is based on the Gaussian process upper confidence bound (GP-UCB) algorithm^44^. The GP-UBC is used in Bayesian optimization problems with an underlying Gaussian processes regression of the form $y_i = f(s_i) + \varepsilon _i$. The difference between our formulation in Eq. (6) and the original GP-UCB is that the latter uses the *mutual information* between the observations $y_i$ and the process *f*^42^, as opposed to the joint differential entropy of $\mathbf{f} _{{\mathscr {A}}}$ only. The mutual information between $y_i$ and *f* is theoretically a better approach. However, the assumption of Binomial outcomes that depend on a transformation of *f*, makes this quantity harder to compute. On the other hand, the use of differential entropy showed satisfactory results in our simulation studies, as shown below.

### Experimental simulation

**Figure 4:**
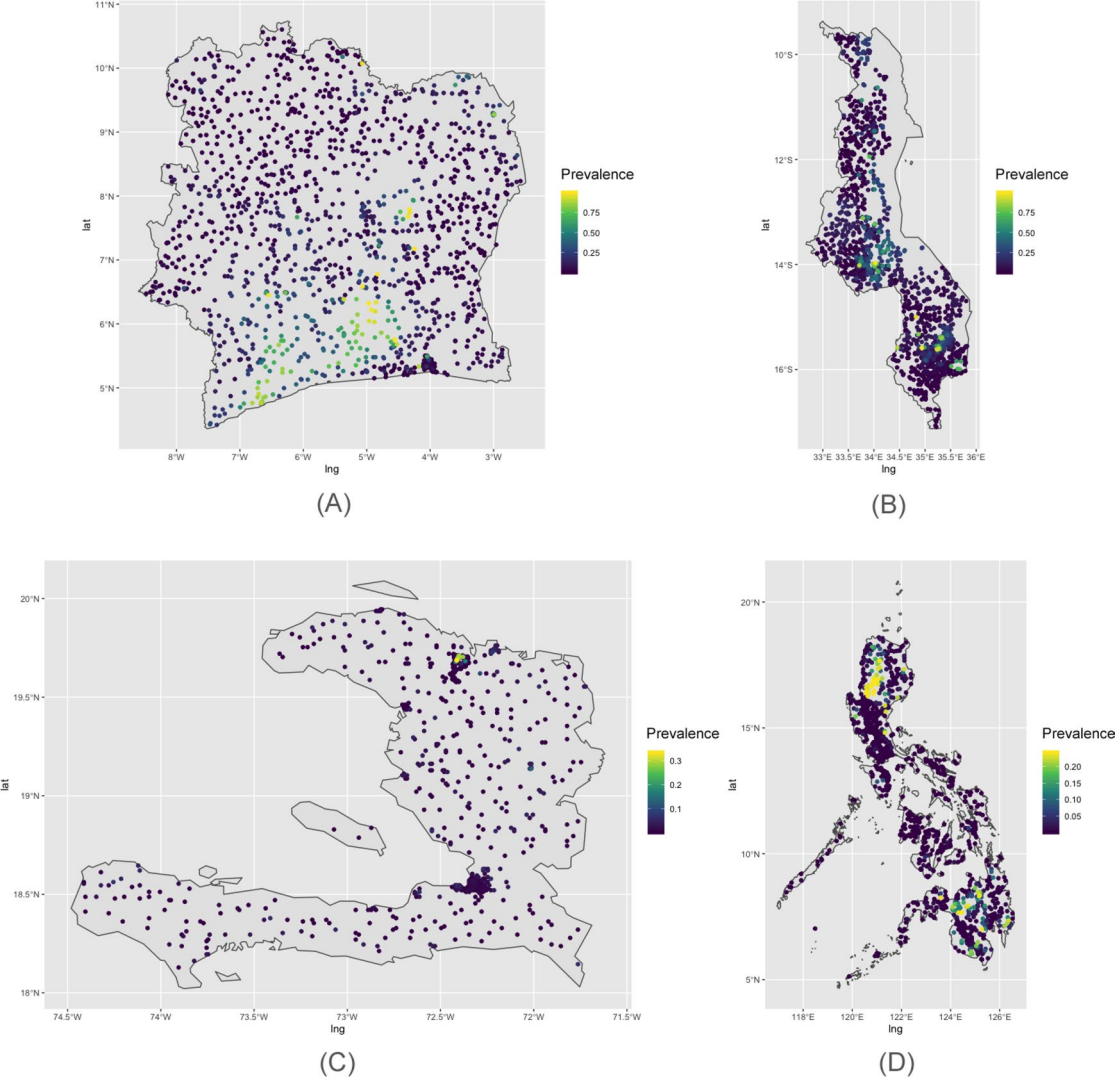
Simulated prevalence scenarios. The locations of the villages is marked by the dots, whose colors represent the hypothetical prevalence of each scenario. (**A**) Côte d’Ivoire (schistosomiasis). (**B**) Malawi (schistosomiasis). (**C**) Haiti (lymphatic filariasis). (**D**) Philippines (lymphatic filariasis).

To test the proposed adaptive spatial sampling approach, we conducted a series of experimental simulation studies parameterized using data from NTD surveys across multiple diseases and countries. We created different scenarios in which the task was to adaptively select new sampling locations with the goal of classifying sites as hotspots and not hotspots. In this procedure, our benchmark was the prediction performance when selecting batches of sampling sites randomly without adaptation. We defined four prevalence scenarios based on cross-sectional prevalence survey data of schistosomiasis from Côte d’Ivoire and Malawi and lymphatic filariasis from Haiti and Philippines (see Supplementary Information). In each of the four countries, we used a universe of up to 1500 candidate survey sites identified with the *Village Finder* algorithm (see Supplementary Information). This algorithm uses gridded population estimates of 2015 from Worldpop to identify clusters of populated places^47^. For computational reasons, if this resulted in more than 1500 clusters for any given country, we randomly sampled 1500 to obtain a final set of candidate locations. Figure 4 shows the cluster locations in each country and the simulated prevalence used as the *truth* during these experiments.

In order to have consistency in our results, we repeated our experiments 50 times per country. In each replicate, we randomly selected 100 locations from the universe of clusters and used them as the locations of the initial set ${\mathscr {D}}_0$. We ran three versions of the experiments, by sequentially selecting batches of size 1, 10 and 50, until we had incorporated 100 new samples. Given a set of initial sampling locations and batch size, we sampled additional locations either completely at random or adaptively following Eq. (6). At each step we fitted the model described in Eqs. (1) and (2). As environmental variables we used: annual mean temperature, temperature seasonality, annual precipitation and precipitation seasonality^48^, elevation (SRTM) and distance to inland water resampled to the same  1km resolution. After fitting the spatial model on each iteration, we computed four out-of-sample validation statistics to measure performance (see Supplementary Information): accuracy, positive predictive value (PPV), sensitivity and mean squared error (MSE). To compute the validation statistics we fitted the model in Eq. (1) to all the available data at each iteration (i.e. $\cup {\mathscr {D}}_{k=0}^{t-1}$ at step *t*) and made predictions on the villages that had not been visited yet (i.e. ${\mathscr {S}} \setminus \cup _{k=0}^{t-1} {\mathscr {A}}^\star _k$ at step *t*). MSE was computed comparing the predicted prevalence vs the simulated prevalence. To compute accuracy, positive predictive value and sensitivity we first classified the villages as hotspots when $p(\theta _i> \vartheta | {\mathscr {D}}_0) > 0.5$ and compared this classification vs the actual class according to the simulated prevalence. Table 2 shows the algorithm followed to carry on our experiments.

**Table 2:** Experimental procedure.

Pseudo code for experiments		
1	for *rep* in $1\rightarrow 100$:	
2	for *m* in $\{1, 10, 50\}$:	*m* = batch size
3	${\mathscr {A}}_0 \leftarrow$ random selection: ${\mathscr {A}}_0 \subset {\mathscr {S}}$ with $\Vert {\mathscr {A}}_0\Vert = 100$	${\mathscr {S}} =$ all villages
4	${\mathscr {A}}^R_0 = {\mathscr {A}}^A_0 = {\mathscr {A}}_0$	$R/A=$random/adaptive
5	$steps = 100 / m$ + 1	total number of iterations
6	for *t* in $1 \rightarrow steps:$	
7	${y}^{\star}_{i} \sim \text{Binomial}{(100, \theta ({\mathscr{A}^{\star}_{t-1}}))}^{\dagger}$	$\star =\{R, A\}$
8	${\mathscr {D}}^\star _{t-1} = \{{\mathscr {A}}^\star _{t-1}, y^\star , \mathbf{x} ({\mathscr {A}}^\star _{t-1}) \}$	$\mathbf{x}$=environmental data
9	find $p(\theta > \vartheta | \cup _{k=0}^{t-1} {\mathscr {D}}^\star _k)$	
10	compute validation statistics on ${\mathscr {S}} \setminus \cup _{k=0}^{t-1} {\mathscr {A}}^\star _k$	
11	${\mathscr {A}}^R_t \leftarrow$ random selection	
12	${\mathscr {A}}^A_t \leftarrow$ acquisition function Eq. (6)	

We repeated each experiment a hundred times (line 1), for batches of size 1, 10 and 50 (line 2). We started with an initial random sample of 100 locations (line 3) for both random and adaptive methods (line 4). We incorporated subsequent samples until 100 additional sampling locations were added (line 5). For the locations selected to be sampled we simulated the observed positive cases according to a Binomial distribution with prevalence $\theta$ (line 7) and incorporated the environmental data (line 8). We then used the accumulated data to find the probability of exceeding the threshold $\vartheta$ (line 9). Finally we defined a new batch of locations according to a random mechanism (line 11) and to the adaptive sampling method proposed (line 12).

$$^{\mathrm{a}}y^R_i = y^A_i$$ for step $$t=0$$.

Random forest and geostatistical models were fit using the R packages ranger 0.11.2^49^ and spaMM 3.0.0^50^ respectively. All the simulated datasets and code developed as part of this study, including that used to conduct the simulation experiments, is available at https://github.com/disarm-platform/adaptive_sampling_simulation_r_functions. Internal Review Board approval was granted from the University of California, San Francisco (IRB Number: 18-25235).

We are also in the process of developing a user-friendly web application to allow both the hotspot mapping and adaptive sampling algorithms to be run without code.

## Supplementary information

Supplementary information.
