# Supplementary material for: Finding hotspots: development of an adaptive spatial sampling approach
Source: Sci Rep. 2020 Jul 2;10:10939. doi: 10.1038/s41598-020-67666-3 (PMC7331748; doi:10.1038/s41598-020-67666-3)
Supplement: Supplementary file 1 — Supplementary information. [file 41598_2020_67666_MOESM1_ESM.pdf]

Supplementary information

Covariance Structure

In equations (1) and (2), it is the function $f(\mathbf{s}_i)$ the one that encodes the spatial structure. Here we model such spatial structure as an *Matérn* covariance, given by

$$\mathbf{K}(\mathbf{s}_i, \mathbf{s}_j) = \frac{2^{1-\nu}}{\Gamma(\nu)} \left(\frac{\sqrt{2\nu}\|\mathbf{s}_i - \mathbf{s}_j\|}{\ell} \right)^{\nu} K_{\nu} \left(\frac{\sqrt{2\nu}\|\mathbf{s}_i - \mathbf{s}_j\|}{\ell} \right), \tag{7}$$

where ν controls the smoothness of the process, ℓ is a lengthscale parameter and K_ν is a modified Bessel function.

Village Finder

The Village Finder algorithm is accessible via a Shiny app that suggests GPS coordinates of populated sites based on 1km resolution Worldpop gridded population data. A populated site is an area that meets certain size and population criteria and can represent a village, a neighborhood of a crowded city or a large but sparsely populated rural area.

The user specifies the following 3 parameters to define the type of population sites queried:

- maximum area size, above which a region cannot be considered as a unique location;
- upper population threshold, above which a location should be counted as a unique location;
- lower population threshold, below which a region smaller than the maximum area size should not be counted as a populated location.

The algorithm works iteratively. First, any 1km grid cells of the Worldpop raster that adhere to the three parameters are identified and the centroids are kept. The gridded population data, minus those grid cells identified in the first round, are then aggregated by a factor of 2 and any aggregated areas that adhere to the parameters are identified. The centroid of the most populated cell in the aggregated area is then assigned as the village location for that aggregated area. The process continues until all aggregated areas have an assigned centroid or until all thresholds are met.

This app and source code are available from:

- https://disarm.shinyapps.io/ui-village-finder (temporary);
- https://github.com/disarm-platform/fn-village-finder.

Simulation of gold standard prevalence scenarios

For each of the four settings (schistosomiasis in Cote d'Ivoire and Malawi and lymphatic filariasis in Haiti and Philippines) we simulated gold standard prevalence estimates for cluster by fitting a spatial model to observed survey data. For each dataset, we first used a Generalized Additive Model (GAM) to fit thin plate spline (non-linear) relationships with four Worldclim variables (mean precipitation, mean temperature, precipitation seasonality, temperature seasonality), elevation (NASA SRTM) and distance to nearest waterbody (Digital Chart of the World) using mgcv (v1.8-27). We then fitted a variogram to the residuals from each model and conditionally simulated a single realization at all clusters using geoR (1.7-5.2.1). This was added to the predictions from the GAM and an inverse logit was applied to get predictions back on the probability scale. This two step process allowed us to fit complex non-linear relationships with covariates plus a residual spatial effect. To ensure an adequate number of hotspot communities for simulation purposes, the simulated datasets were adjusted slightly to ensure the mean prevalence was roughly equal to the relevant disease specific hotspot prevalence threshold (i.e. 10% for schistosomiasis and 2% for lymphatic filariasis). Supplementary Fig. S1 shows histograms of the cluster level prevalence for each country.

Validation Statistics

To measure the performance of the classification model we used four different metrics. To define them, we first need to define the following terms:

- True positives (tp): cases where the actual category and the predicted category are both positive (e.g. a site classified as a hotspot actually has a prevalence above the threshold of interest).
- True negatives (tn): cases where the actual category and the predicted category are both negative (e.g. a site classified as not being a hotspot actually has a prevalence below the threshold of interest).
- False positives (fp): cases where the actual category is negative, but the predicted class is positive (e.g. the site is classified as a hotspot, but the actual prevalence is below the threshold of interest).

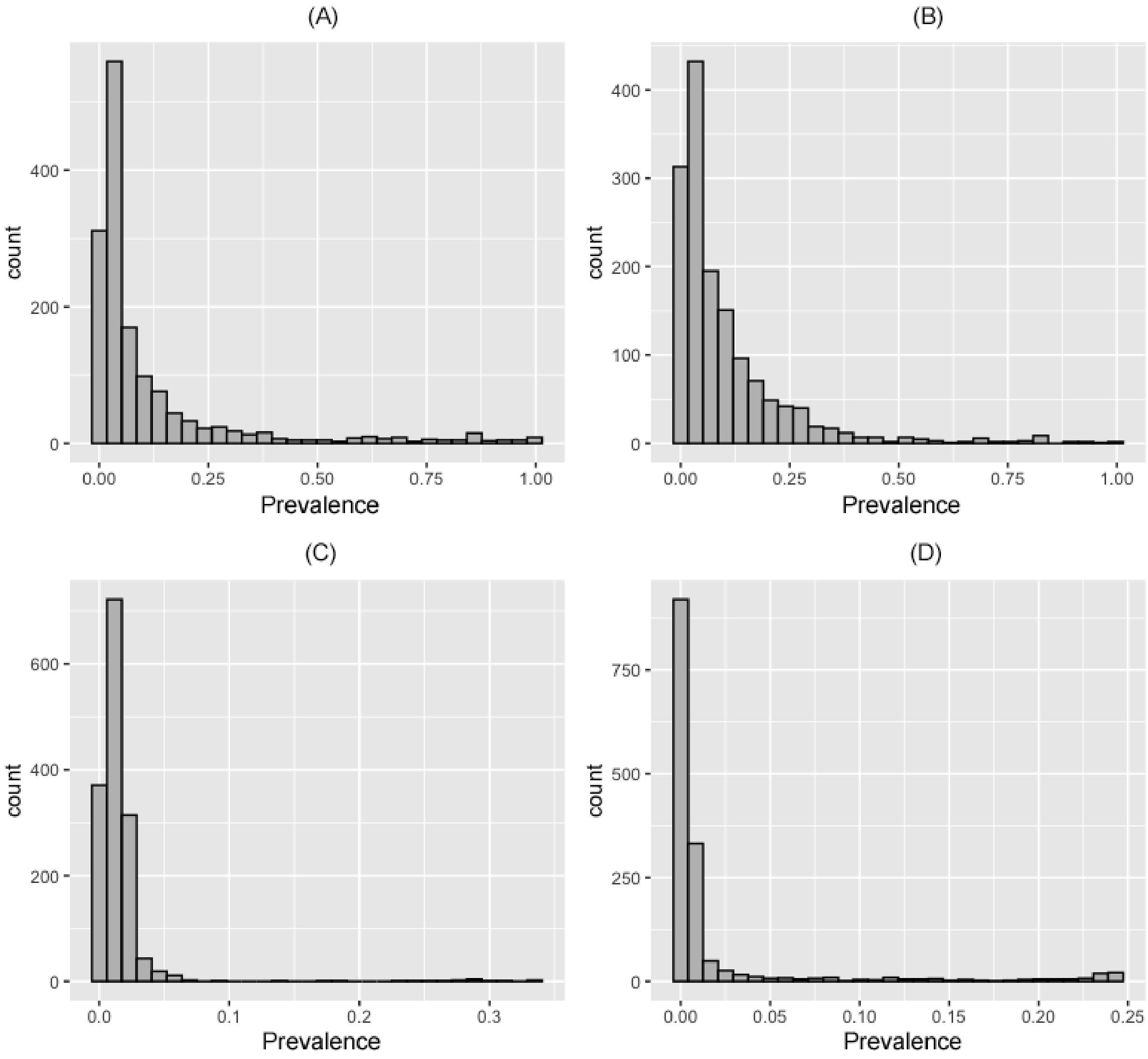

Figure S1. Histograms of the simulated prevalence data by country. (A) Cote d'Ivoire, (B) Malawi, (C) Haiti and (D) Philippines.

- False negatives (fn): cases where the actual category is positive, but the predicted class is negative (e.g. the site is classified as not being a hotspot, but the actual prevalence is above the threshold of interest).

Accuracy. The proportion of sites correctly classified.

$$Accuracy = \frac{tp+tn}{tp+fp+tn+fn}. \tag{8}$$

Positive predicted value. The proportion of sites classified as hotspots that were true hotspots.

$$PPV = \frac{tp}{tp+fp}. \tag{9}$$

Sensitivity. The proportion of true hotspots that were correctly classified.

$$Sensitivity = \frac{tp}{tp+fn}. \tag{10}$$

Mean squared errors. The average of the squared differences between the target value (predicted prevalence) and the observed value (actual prevalence).

$$MSE = \frac{1}{m}\sum_{i}^{m}\left(\frac{y_i}{n_i} - \theta_i\right)^2. \tag{11}$$
